# All-Opto Plasmonic-Controlled Bulk and Surface Sensitivity Analysis of a Paired Nano-Structured Antenna with a Label-Free Detection Approach

**DOI:** 10.3390/s21186166

**Published:** 2021-09-14

**Authors:** Sneha Verma, Souvik Ghosh, B.M.A. Rahman

**Affiliations:** School of Mathematics, Computer Science and Engineering, University of London, London EC1V 0HB, UK; souvik.ghosh.1@city.ac.uk (S.G.); B.M.A.Rahman@city.ac.uk (B.M.A.R.)

**Keywords:** surface plasmon resonance, sensitivity, elliptical nanoantenna, Full-Width at Half-Maximum (FWHM), Figure-Of-Merit (FOM), plasmonic wavelength, bulk sensitivity, surface sensitivity

## Abstract

Gold nanoantennas have been used in a variety of biomedical applications due to their attractive electronic and optical properties, which are shape- and size-dependent. Here, a periodic paired gold nanostructure exploiting surface plasmon resonance is proposed, which shows promising results for Refractive Index (RI) detection due to its high electric field confinement and diffraction limit. Here, single and paired gold nanostructured sensors were designed for real-time RI detection. The Full-Width at Half-Maximum (FWHM) and Figure-Of-Merit (FOM) were also calculated, which relate the sensitivity to the sharpness of the peak. The effect of different possible structural shapes and dimensions were studied to optimise the sensitivity response of nanosensing structures and identify an optimised elliptical nanoantenna with the major axis *a*, minor axis *b*, gap between the pair *g*, and heights *h* being 100 nm, 10 nm, 10 nm, and 40 nm, respectively. In this work, we investigated the bulk sensitivity, which is the spectral shift per refractive index unit due to the change in the surrounding material, and this value was calculated as 526–530 nm/RIU, while the FWHM was calculated around 110 nm with a FOM of 8.1. On the other hand, the surface sensing was related to the spectral shift due to the refractive index variation of the surface layer near the paired nanoantenna surface, and this value for the same antenna pair was calculated as 250 nm/RIU for a surface layer thickness of 4.5 nm.

## 1. Introduction

Nanostructures have recently gained much popularity due to their many applications and have established a new perspective of developing nanotechnology for optical biosensing applications. A nano-shaped antenna can provide an excellent opportunity for molecular biosensing applications due to its efficient Localised Surface Plasmon Resonance (LSPR) [1]. The LSPR maintains strong electromagnetic field confinements, which makes it an efficient candidate for accurate sensing applications such as biomedical sensing, biomedicines, communications, solar cells, imaging, energy harvesting, water quality control, disease diagnosis and prevention, or improvements. Nanotechnology has experienced remarkable popularity in recent years because of the highly efficient performances such as in absorption, scattering, extinction, and transmission/reflection at the nanoscale; however, there are many potentials still left to be explored. In this work, a novel paired gold elliptical nanostructure is proposed, and here, the Drude–Lorentz model was used to calculate the dielectric constant of a metal with free electrons.

Even though the principle of near-field microscopy was proposed by Synge in 1928, this was not further explored because of the fabrication limit at that time. In 1973, Bailey and Fletche received a patent for electromagnetic wave converters, which was the first report of a nanoantenna that is similar to modern-day nanoantennas [2]. Later on, in 1985, Wessel reported an idea for high electric field confinement due to tiny nanometallic particles, which can be visualised with the help of scanning microscopy [3], and discussed the importance of the surface plasmon resonance of these nano-sized particles. In 1989, Alvin M. Marks reported a super-submicron electron beam writer for direct conversion of light into electric current [4]. That article discussed the effect of the different shapes and parameters of the plasmonic antenna for a variety of applications. In 2004, Atay et al. [5] synthesised circular periodic arrays of gold antennas with strong resonance and far-field patterns. Similarly, Lahiri et al. designed asymmetrically split ring resonators for the detection of biological materials [6]. Nanoantennas are used for Surface-Enhanced Raman Spectroscopy (SERS), which have the advantage of resonating simultaneously both in the infrared and visible regions [7]. Additionally, paired nanoantennas of different shapes such as bow-tie-shaped [8,9,10,11], nanodiscs [12,13,14], nanorods [10,15,16,17], and elliptical nanoantennas have been reported [18,19] for the visible region. In 2008, Fisher and Martin reported [20] a sensitivity of 500–510 nm/RIU for a bow-tie-shaped plasmonic nanoantenna with a 10 nm gap between the sharper 20 nm tips. In the same year, Anker et al. [21] reviewed the effect of the shape and size of plasmonic nanoantennas, as well as the sensing by surface-enhanced Raman spectroscopy. In 2011, Sage et al. [22] reported the advances in the localised SPR for spectroscopy-based biosensing using triangular Ag nanoparticles with a thickness of 10 nm. They functionalised multiple spectrally distinct nanoparticles by changing the material, shape, and size of the constituent antennas to target different species for multiplexing. Recently, Chao et al. [23] reported a higher sensitivity of 1120 nm/RIU using a metal–insulator–metal bus waveguide side coupled to a ring resonator comprised of many Ag nanorods. More recently, Armstrong et al. [24] and Mauriz and Lechuga [25] reviewed the rapid advancement of plasmonic biosensors for single-molecule biosensing.

Recently, there have been many reports of biosensors using artificially created metamaterials. In 2009, Kabashin et al. reported [15] a sensitivity of 30,000 nm/RIU for two-dimensional porous gold nanorod arrays on a plasmonic hyperbolic metamaterial for biosensor applications. However, despite being miniaturised for commercial biosensing applications, this bulk Kretschmann arrangement is not suitable for more compact integrated photonic sensors. Subsequently, in 2016, Sreekanth et al. [26] reported a similar sensitivity of 30,000 nm/RIU for a grating coupled hyperbolic metamaterial for bulk refractive sensing. Although the device, compared to [15], was further miniaturised and also multiplexed, it still utilised the bulk plasmonic modes and additionally needed more expensive fabrication and excitation techniques. In the same year, the same group also reported [27] a similar structure with a high angular sensitivity of 7000 degrees per RIU. In 2017, Lee et al. [28] reviewed the use of metamaterials and metasurfaces for sensor applications. More recently, Garoli et al. [29] reported a possible low-cost fabrication and simple excitation techniques using nanoporous gold metamaterials and achieved a sensitivity of 15,000 nm/RIU. The above discussions show the promising results of strong resonance and field confinement through numerical modelling and experimental investigations. Recently, the numerical design and simulation of gold nanoantennas was modelled by a computationally efficient Finite Element Method (FEM) [8,11,18,19,30,31] and the versatile Finite Difference Time Domain method (FDTD) [32,33,34,35,36].

This work is organised as follows. Section 2 describes the numerical methods and model optimization with two subsections containing the performances of the optimised nanostructure and a comparison of the optimised structure with the published work. The paper is concluded in Section 3. Finally, the novelties of the proposed work are described in Section 4.

## 2. Numerical Methods and Model Optimization

**Figure 1:**
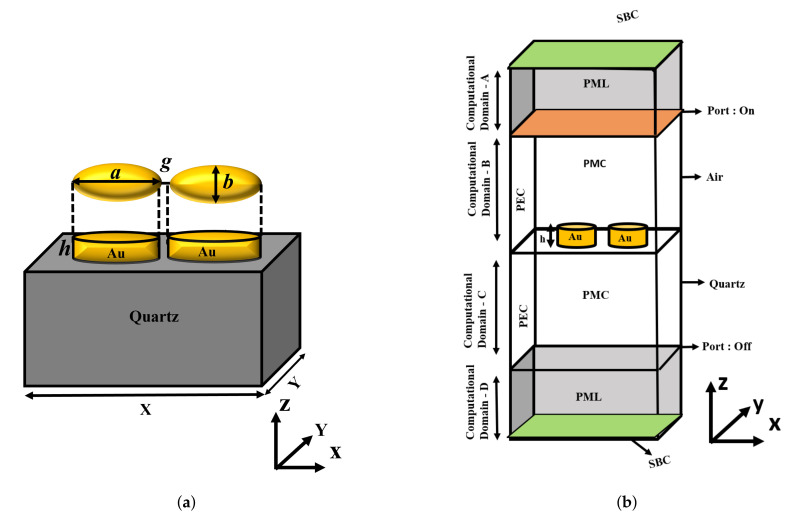
Designed model in COMSOL Multiphysics: (**a**) Top and side views of a paired elliptical gold nanoantenna. (**b**) Computational model of the paired gold nanoantenna along with the boundary conditions.

In this work, an FEM-based frequency domain approach [8,11,18,19,30,31] was used to determine the plasmonic field distribution of gold nanoparticles. Figure 1 shows the dimensions of the nanostructures, where *a* and *b* are the major and minor axes, *h* is the height, and the separation gap is *g*.

The schematic diagram of a paired gold nanostructured antenna on a quartz substrate is shown in Figure 1a, the unit cell of the quartz substrate on which a paired gold nanostructure was placed. All the numerical simulations were carried out by using COMSOL Multiphysics based on the FEM. This figure shows that the electronic transitions in metal are directly proportional to the complex dielectric constant, and the electric and magnetic fields are connected by this permittivity to maintain the accuracy in the computational results. The Drude–Lorentz model was used to obtain the property of gold, because it focuses on the free electron present in metals, which causes surface plasmon resonance. The Drude free electron model has been proven to be more suitable for materials that have more free electrons as compared to bound electrons. The calculated real and imaginary parts of the dielectric constant are also shown in Figure 1a, which were used to calculate the transmission and reflection coefficients. To reduce the simulation time, we simulated one unit cell, enforcing periodic boundary conditions, as shown in Figure 1b. A metal disk was excited by x-polarised light in the z-direction from the top with wave excitation on, and additionally, Scattering Boundary Conditions (SBCs) were placed at the bottom and top of the computational domain. The entire numerical problem was solved in the frequency domain to obtain the scattered field distributions and the transmission and reflection spectra. In the FEM solver, the entire structure was discretised into “study” mesh elemental size. Additionally, a Perfectly Matched Layer (PML) with a 200 nm height on the top of the air domain and the bottom of the quartz substrate was introduced to avoid the artefacts of back reflection in the simulations. The periods along the x- and y-directions were taken as 400 nm and 200 nm, respectively. The height of the metal disks was initially taken as 40 nm from the surface of the quartz substrate. A Perfect Magnetic Conductor (PMC) and Perfect Electric Conductor (PEC) were used along the x- and y-directions, respectively, to enforce the periodicity of the structure. In this article, the sensitivity study of four different designs, single sphere and single disk, paired disk, and elliptical antenna, are presented. In order to investigate the physical plasmonic properties, the conventional Maxwell equation was solved by using the FEM considering the harmonic dependence of the electric field $E\left(r,t\right)=E\left(r\right)e^{-jwt}$. We used the Helmholtz equation throughout the computation, which can be derived from the conventional Maxwell equations, as shown below,

(1) $$\nabla^{2}E+k_{0}^{2}\varepsilon E=0$$

where $k_{0}$ is the wavevector. Here, a time harmonic propagating field was considered with $E\left(x,y,z\right)=E\left(x,y,z\right)e^{j\beta z}$, where β is the propagation constant. In complex form, γ=α+jβ, and when α=0, then γ=jβ, representing the propagation dependence in the *z*-direction. An x-polarised wave in the z-direction was launched from the top surface to excite the antenna, which generated the LSPR after the interaction with the proposed gold nanoshapes. The sensitivity (nm/RIU) *S* of the designed structure is defined as the ratio of the resonant wavelength $\lambda_{res}$ shift with the change in the environmental refractive index $\delta n_{s}\left(RIU\right)$:

(2) $$S=\frac{\delta \lambda_{res}\left(nm\right)}{\delta n_{s}\left(RIU\right)}$$

**Figure 2:**
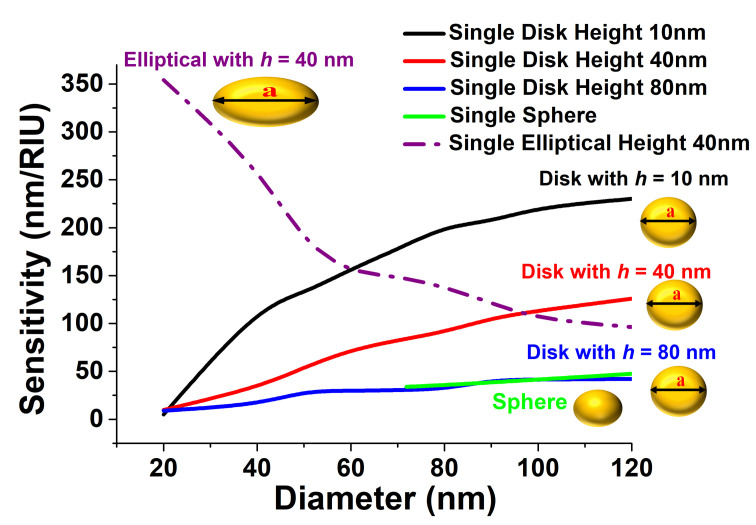
Sensitivity variation with the diameter of a single nanodisc at a 10 nm, 40 nm, and 80 nm height and a sphere and single elliptical disk when *a* = 100 nm, *b* = 10 nm to 100 nm, and *h* = 40 nm.

The sensitivities of a single disk and a sphere are shown in Figure 2. Here, the green curve depicts the sensitivity values of a single sphere of different diameters placed on a quartz substrate, and this value was approximately 25 nm/RIU for *d* = 70 nm–120 nm. These single spheres can be used to detect the target in complex media such as serum because of the amplitude variation and shift in the plasmonic wavelength, as discussed by Chen et al. in 2010 [37], and the response of the dimers and trimers of spheres were also shown by Deep et al. in 2015 [38]. To study the effect of the height and diameter of a single disk, the sensitivity of a single disk is also shown by the blue curve when its height was 80 nm. It can be observed that its sensitivity decreased as the diameter was reduced, and for such a thicker disk, its value was comparable to that of the sphere for a similar diameter. However, as the height was further reduced, the sensitivity significantly increased. The sensitivity values of a single disk of a 40 nm height is shown by the red curve, which almost saturated to 125 nm/RIU when the diameter was above 100 nm. When the height was further reduced to 10 nm, shown by the black curve, it can be clearly observed that the sensitivity of a circular disk increased to 225 nm/RIU as its diameter was increased to 120 nm, and then, it saturated.

Additionally, the sensitivity of the single elliptical disk is shown by the purple dashed-dotted curve. Here, its major axis, *a*, was kept constant at 100 nm and its minor axis, *b*, was varied. It can be observed that as its minor axis *b* was reduced, the sensitivity rapidly increased and reached up to 350 nm/RIU when *b* = 10 nm. This demonstrates a very important opportunity, that the sensitivity of a noncircular elliptical disk can be significantly increased by reducing its overall dimensions, unlike the very limited sensitivity that can be achieved by a larger circular disk. It was clearly observed that the sensitivity depended both on the diameter, the major and minor axes, and the thickness of the disks, but to make a fair comparison, a thickness of 40 nm is used in the following section for all the cases, which can also be reliably fabricated.

**Figure 3:**
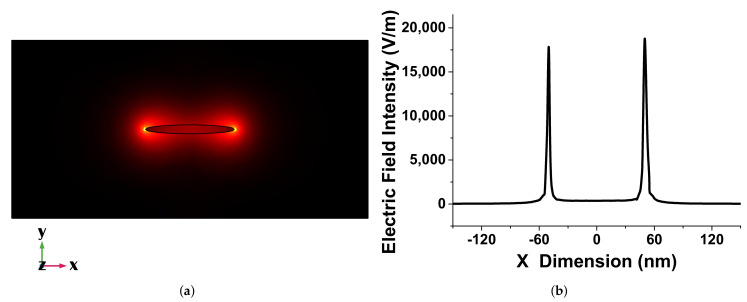
(**a**) Ex mode field profile of a single elliptical disk antenna when *a* = 100 nm, *b* = 10 nm, and *h* = 40 nm. (**b**) Electric field variation along the x-axis for a single elliptical disk when *a* = 100 nm, *b* = 10 nm, and *h* = 40 nm.

Figure 3a shows the strong electric field confinement at the sharp corners of the elliptical disk. This clearly demonstrates that, due to the lack of a circular symmetry, the field was more localised. Variation of the electric field $E_{x}$ in the x-dimension through the centre of the single elliptical disk is shown in Figure 3b. This figure also shows a strong electric field intensity up to 18,000 V/m at the corners of the single elliptical disk with *a* = 100 nm, *b* = 10 nm, and *h* = 40 nm. This value is nearly double that of a circular disk, with *a* = *b* = 100 nm, and *h* = 40 nm. Due to its strong electric field confinement, a single elliptical disk can be considered as a good candidate for sensing applications as compared to a single circular disk.

### 2.1. Paired Elliptical Nanoantenna

**Figure 4:**
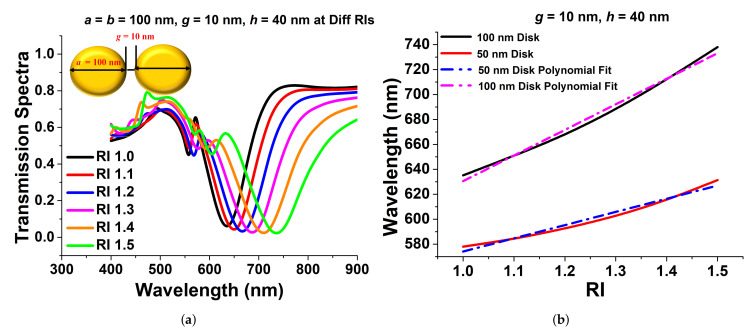
(**a**) Transmission spectra of a paired 100 nm circular nanodisc with a 10 nm separation distance and a 40 nm height. (**b**) Sensitivity and R-squared error calculation of a 100 nm and a 50 nm paired circular disk.

It is well known that a paired disk, which is also called a dimer, can enhance the field intensity in the gap, so next, both paired circular and elliptical disks were evaluated. Hence, a paired circular disk on the quartz crystal was studied, and the transmission spectra of a 100 nm gold nanodisc surrounded by a material of different refractive indices are shown in Figure 4a. Here, it can be clearly observed that as the refractive index increased, the resonating wavelength shifted towards a higher range. Here, the height of the disk, *h*, and the separation gap, *g*, were taken as 40 nm and 10 nm, respectively. Figure 4b shows that the resonating wavelength increased as the surrounded refractive index, RI, increased. The sensitivity of the paired circular nanodisc was calculated from the slopes of these two curves as 105.79 and 205.18 for diameters of 50 nm and 100 nm, respectively. We also show that a nearly linear relationship between the wavelength change and the RI variation existed. The R-squared error was calculated as 0.98915 and 0.9137 for a 50 nm and a 100 nm diameter, suggesting an almost linear relationship. In Figure 4b, it is clearly shown that at a higher diameter, the 100 nm disk, the achieved sensitivity was higher than that for 50 nm.

**Figure 5:**
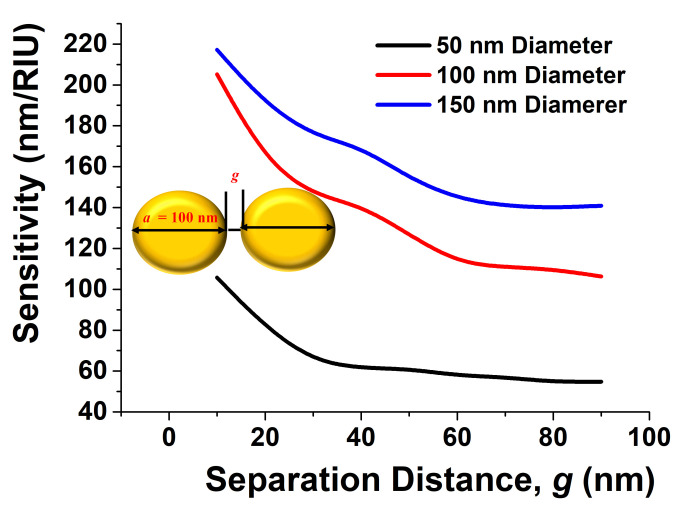
Sensitivity analysis of paired circular nanodiscs of a 50 nm, a 100 nm, and a 150 nm diameter with different separation distances.

As sensitivity also depends on the separation between the circular paired disk, next, its effect was studied, and the sensitivities of a 50 nm, a 100 nm, and a 150 nm disk diameter with the separation distance are shown in Figure 5. It can be noted that when the separation distance between the disks, *g*, was large, the sensitivity was similar to that of a single disk, whereas it was clear that as the separation distance, *g*, was reduced, and the sensitivity increased. Therefore, it was demonstrated here that the sensitivity of the paired disk was always higher than that of an individual disk. Zhao et al. reported Au nanoparticles on a microsphere structure with *g* as small as 0.82 nm [39]. It was also shown here that as the diameter *a* of the disk increased, its sensitivity also increased.

**Figure 6:**
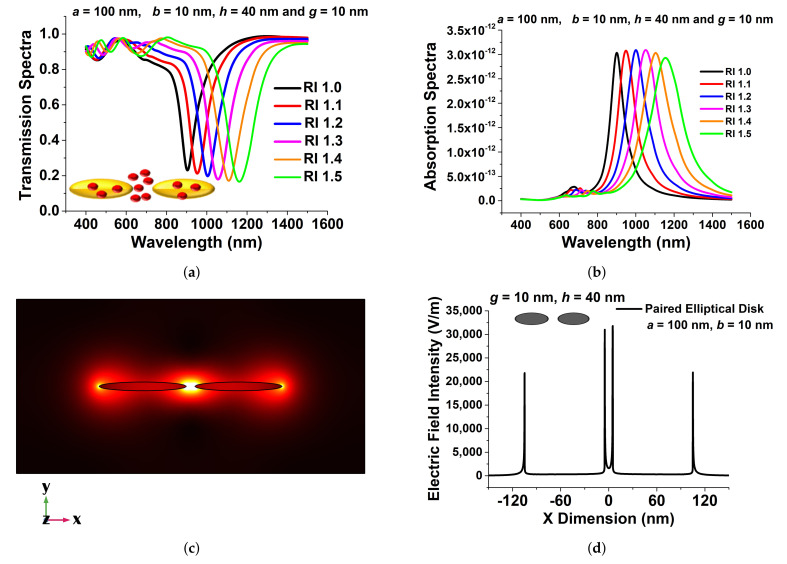
(**a**) Transmission spectra of the optimised paired elliptical antenna of 100 nm, with a 10 nm major and minor axis, respectively, 10 nm separation distance, *g*, and a 40 nm height, *h*. (**b**) Absorption spectra of the same structure. (**c**) *E^x^*, the mode field profile of the optimised paired structure. (**d**) Comparison of the electric field distribution of the optimised elliptical disk and the 100 nm paired circular nanodisc at a 10 nm separation distance, *g*, and a 40 nm height, *h*.

Next, to explore the sensitivity of an elliptical paired nanodisc, its minor axis *b* was varied from 10 nm to 100 nm, keeping the major axis *a* fixed at 100 nm. The height of the antenna was fixed at 40 nm. These paired nanostructures were excited with linearly x-polarised light in the z-direction with principal polarization where the electric field was parallel to the major axis *a*. The sensitivity was optimised from the transmission spectra at different refractive index values. The transmission spectra for the selected design with different surrounding media (n) are shown in Figure 6a. Here, the major axis *a*, minor axis *b*, separation distance *g*, and height were taken as 100 nm, 10 nm, 10 nm, and 40 nm, respectively. Since the design showed a more efficient shift in resonating wavelength, it can be used as a refractive index sensor and can be a strong candidate for RI sensing applications. To detect targeted media in the IR region, the spectral absorption of the narrowband paired structures can also be tailored to match the characteristic absorption spectra of the targeted RI.

Figure 6b shows the the absorption spectra of the designed paired nanoantenna array for six targeted RIs, which supported the observed transmission spectra and showed accurate responses. Figure 6c shows the electric field intensity in the separation region and very high electric field confinement at the centre of the paired antenna. For label-free detection, the strength of the localised electric field plays an important role. In the designed structure, as the strongest electric field occurred at the middle of the separation gap, such an ultra-strong electric field can be exploited for sensing applications. Variation of the electric field $E_{x}$ in the x-direction through the centre of the antenna pair is shown in Figure 6d for circular and elliptical pairs. Since electron conduction produced an efficient force at the surface of the paired structure, this resulted in a substantial enhancement of the electric field in the separation gap region, as seen in Figure 6d. From this figure, for *a* = 100 nm, *b* = 10 nm, *g* = 10 nm, and *h* = 40 nm, the maximum electric field, shown by the black curve in Figure 6d, reached up to 35,000 V/m at the inner edge of the paired elliptical disks, which clearly showed that the maximum field intensity was more then 50% higher in the gap compared to the field at the outer edges. Due to its higher strength of electric field confinement, it can be considered as a better candidate for sensing applications. From the electric field profile, it can be concluded that for a paired structure, a significant field enhancement can be observed at sharp corners by reducing the minor axis *b*. The LSPR enhancement occurred as a result of this coupling, as the elliptical structures interacted more strongly when they came closer to each other. The shift in the transmission and absorption spectra of the resonating wavelength was smaller when the separation distance was higher, so in order to achieve a strong electric field confinement, a smaller separation distance is preferred. It is clear from the above figures that the maximum localised field intensity formed due to the coupling of surface waves on the nanostructures, and it can be an excellent candidate for RI sensing applications; this quite sharp pattern can be helpful for scattering controlled EM waves such as label-free RI detection by surface-enhanced Raman scattering and many more point-of-care applications.

### 2.2. LSPR Sensing Calculation of the Optimised Structure

**Figure 7:**
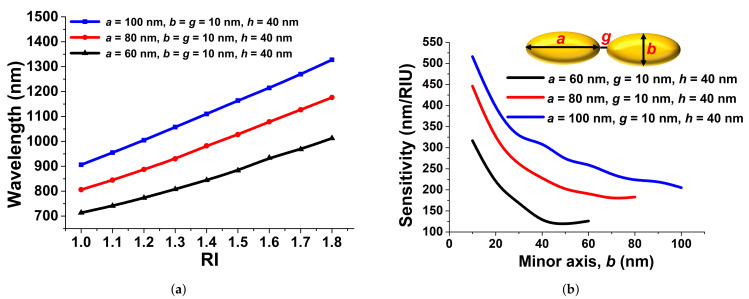
(**a**) Polynomial fit when the major axes a were at 100 nm, 80 nm, and 60 nm at an RI from 1.0 to 1.8. (**b**) Sensitivity comparison when the major axes a were at 100 nm, 80 nm, and 60 nm at different minor axes *b* with a 10 nm separation gap, *g*.

For a good sensor, a higher sensitivity is the key design objective, but the sharpness of the transmission and reflection curve is also important for easy detection. In this particular section, we focus on the important device parameters such as the Sensitivity (nm/RIU), the Full-Width at Half-Maximum (nm), the figure-of-merit, and the R-squared error. Figure 7a shows the variation of the resonating wavelength with the refractive index for three different major axis *a* values. Here, its sensitivity (nm/RIU) was calculated by a polynomial linear fit of the sensitivity analysis, and for 60 nm (shown by the black curve), its slope, *S*, was calculated as 320.02 nm/RIU with a 0.9974 R-squared error. However, for 80 nm, the *S* was calculated to be slightly higher than that for *a* = 60 nm at 467.69 nm/RIU with the associated 0.9985 R-squared error, shown by the red curve. Finally, the sensitivity, *S*, was calculated 526.12 nm/RIU for *a* = 100 nm with a 0.9996 R-squared error, as shown by the blue curve in Figure 7a.

**Figure 8:**
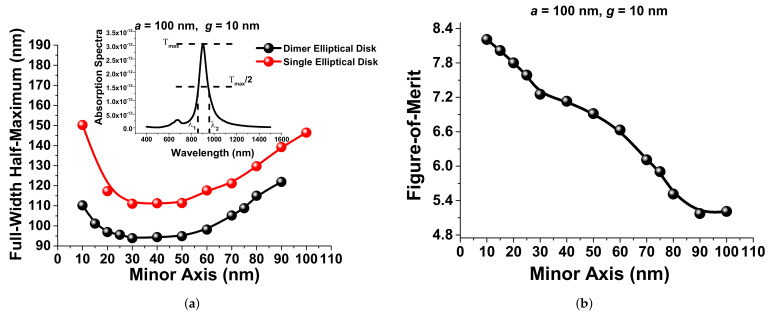
(**a**) Variations of the FWHM with the different minor axes, *b*, when the major axes and separation gap were fixed at 100 nm and 10 nm, respectively. (**b**) Comparison of the Figure-Of-Merit (FOM) calculation at different minor axes when the major axes and gap were fixed at 100 nm and 10 nm, respectively.

The variation of the sensitivity, *S*, with the minor axis, *b*, for three fixed values of *a* = 60 nm, 80 nm, and 100 nm with *g* = 10 nm is shown in Figure 7b. It can be seen that as the major axes, *a*, increased from 60 nm to 80 nm and 100 nm, the sensitivity, *S*, increased, as shown by the blue, red, and black curves, respectively, in Figure 7b. Additionally, it is clearly shown that as the minor axis, *b*, decreased, the sensitivity increased for the fixed major axes, *a*. For *a* = 60 nm, when the minor axis *b* = *a*, its sensitivity, *S*, reached approximately 125 nm/RIU, the same for a paired circular antenna with *b* = *a* = 60 nm, but as the minor axis, *b*, was reduced, the sensitivity increased exponentially and reached nearly 320.02 nm/RIU when *b* = 10 nm, shown by the black curve. For *a* = *b* = 80 nm, the sensitivity was calculated as around 170 nm/RIU, and it gradually increased up to 450 nm/RIU when *b* = 10 nm, shown by the red curve. However, when *a* = *b* = 100 nm, the sensitivity, *S*, was observed to be nearly 250 nm/RIU and exponentially increased up to 520 nm/RIU when the minor axis *b* = 10 nm, shown by the blue curve. This shows that very high sensitivity can be achieved by decreasing the minor axis, *b*, for a large major axis, *a*. Measurement accuracy also depends on the sharpness of the resonance curves, and this can be quantified by the FWHM, which is defined as the difference between the two wavelengths, where the response is half of its maximum values:

(3) $$FWHM=\lambda_{1}-\lambda_{2}$$

where $\lambda_{1}$ and $\lambda_{2}$ are the wavelengths, when the transmission is half of the maximum value, shown as an inset in Figure 8a.

The comparison of the FWHM for a single and a paired elliptical disk is shown where the FWHM of the single elliptical disk initially reduced as the minor axis *b* was reduced and reached its minimum value of 120 nm for *b* between 30 nm and 40 nm; after that, it increased, as shown by the red curve in Figure 8a. The same trend follows for the paired elliptical disk, where as the minor axes *b* were reduced, initially, the FWHM decreased, reaching a minimum value of 95 nm for a minor axis *b* between 30 nm and 40 nm; after that, it increased, as shown by the black curve in Figure 8a. As a result, we can evaluate different designs considering the FWHM values also. As in the presented work, we preferred to have a high sensitivity *S* and also a sharper resonance (that is, a smaller FWHM), the Figure-Of-Merit (FOM) can be considered as an important parameter for the design of a paired nanoantenna array. The FOM can be defined as the ratio of the sensitivity to the FWHM:

(4) $$FOM=\frac{S(nmRIU^{-1})}{FWHM}$$

The variation of the FOM with the minor axis *b* is shown in Figure 8b. In these cases, the major axis *a*, the separation distance *g*, and the height *h* were fixed at 100 nm, 10 nm, and 40 nm, respectively. Shreekanth et al. [26] also considered a similar FOM, but instead of the FWHM in terms of Δλ, they considered Δω. It can be observed that, when the minor axis *b* was reduced below 30 nm, even though the sensitivity increased exponentially (shown in Figure 7b), the FOM increased only moderately, since the FWHM was rather increased in this case.

**Figure 9:**
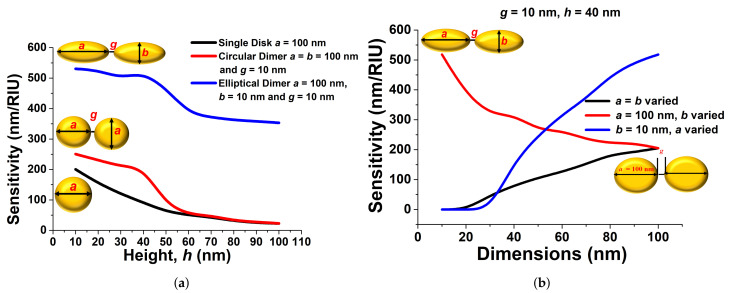
(**a**) Sensitivity comparison of single, double disk, and optimised paired elliptical nanoantenna with a 10 nm separation gap and a 100 nm major axis. (**b**) Sensitivity comparison of the variation of the major axes *a*, minor axes *b*, and the paired disk variable.

Figure 9a shows the sensitivity variation for single and paired circular and elliptical disks. It can be noted that as the height *h* of the antenna was reduced, *S* increased for all the cases. However, the *S* of the paired circular disk (shown by the red curve for *a* = *b* = 100 nm) was higher than that of a single disk (shown by a black curve for *a* = *b* = 100 nm), in particular for a lower antenna height. However, the sensitivity of an elliptical paired disk (shown by the blue curve, with *a* = 100 nm, *b* = 10 nm) showed the highest values mainly with smaller heights. Figure 9a also shows that the sensitivity remained nearly constant when the antenna height *h* was greater than 60 nm. Figure 9b shows the sensitivity analysis for circular and elliptical paired disks. In these case, their height *h* and separation gap *g* were taken as 40 nm and 10 nm, respectively. The sensitivity of a circular disk (with *a* = *b*) increased as its diameter increased and reached around 220 nm/RIU when *a* = *b* = 100 nm.

Our results compared well with the sensitivity reported by Tsai et al. [40] for a circular dimer; however, it can be noted that they considered a much larger disk. The sensitivity variation of a coupled elliptical disk with the minor axes *b* is shown by the red curve, when its major axis *a* was fixed at 100 nm. Although, for a circular paired disk, its sensitivity can be increased by increasing its size, thus larger axes, for an elliptical disk pair, it is shown here that its sensitivity can rather be increased by reducing the minor axis *b*, shown by the red curve. A very high sensitivity of 526–530 nm/RIU was achieved with a 10 nm minor axis *b*, so we fixed it for the further observations. The blue curve shows the sensitivity of a paired elliptical disk, when its minor axis *b* was fixed at 10 nm and its major axis *a* was varied. Here, it can be noted that a higher sensitivity of 526–530 nm/RIU could be achieved when *a* = 100 nm, which is the same value of the left-most point of the red curve. Here, as *a* was reduced, the sensitivity decreased, as the asymmetry was reduced. We analysed the various structural measurements with full-wave computational simulations, then implemented our observations to optimise the periodic paired nanoantenna designs to explore their use in biosensing applications. The simulation results presented here validated the usefulness of the design approaches used to optimise the sensor sensitivity. Finally, in this paper, we showed that a paired nanoantenna with a sensitivity of 526–530 nm/RIU and an FWHM and FOM of 108.86 nm and 8.19 $RIU^{-1}$ can be obtained for a coupled elliptical disk with *a* = 100 nm, *b* = 10 nm, *h* = 40 nm, and *g* = 10 nm. The sensitivity attained can be applied to innovative biomedical imaging modalities, as well as biosensors. Tsai et al. also [40] reported that, by using a paired nanoring, the sensitivity can be increased up to 50%, but our work showed that for an elliptical dimer, the sensitivity can be increased by more then 100%, yet using a much smaller overall size of the antenna.

## 3. Surface Sensing Outcomes of the Paired Elliptical Nanostructured Antenna

Finally, for label-free sensing, the surface sensitivity was also calculated for the optimised structure by measuring the confinement of the electric field close to the nanoantenna. The enhanced electric field intensity along the edges and gap had a significant impact on the surface sensitivity. The presence of the target can change either the refractive index or thickness or both of the sensing layer.

**Figure 10:**
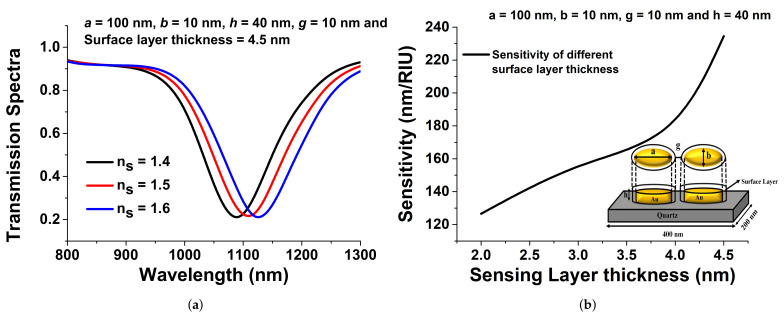
(**a**) Transmission spectra for surface sensing of the optimised paired elliptical antenna disk when *a* = 100 nm, *b* = 10 nm, *h* = 40 nm, *g* = 10 nm, and surface layer thickness = 4.5 nm. (**b**) Surface sensitivity analysis of the optimised elliptical disk when *a* = 100 nm, *b* = 10 nm, and *h* = 40 nm.

The transmission spectra of the surface sensing are shown in Figure 10a when the refractive index ($n_{s}$) of a 4.5 nm-thick sensing layer was varied for *a* = 100 nm, *b* = 10 nm, *h* = 40 nm, and *g* = 10 nm. From this figure, it can be seen that as the RI of the surface layer increased from 1.4 to 1.6, the resonating wavelength shifted towards a larger resonating wavelength, as shown by the black, red, and blue curves. Figure 10b shows the sensitivity response at different surface thicknesses when *a* = 100 nm, *b* = 10 nm, *h* = 40 nm, and *g* = 10 nm. For all the sensitivity analysis, the RI of the surrounding medium was fixed at 1.33 for water, but the refractive index of the surface layer, $n_{s}$. Figure 10b illustrates that as the surface layer thickness increased, the corresponding sensitivity increased and reached up to 240 nm/RIU.

The sensitivity and FWHM of the proposed paired structure were also compared to the published work and are shown in Table 1. The sensitivity of the proposed structure was higher compared to the previously published results. The comparison of the published results of different dimensions with our new approach of the designed structure showed that our approach had a good sensitivity and FWHM; this shows that our findings are quite promising as compared to the published results. The selected parameters (sensitivity (nm/RIU), FWHM (nm), and FOM) can be good parameters for describing the sensor performance and can be used to evaluate different properties in order to improve its efficiency.

## 4. Conclusions

Gold nanoantennas have been used in a variety of biomedical applications due to their attractive electronic and optical properties, which are shape- and size-dependent. In this work, the bulk and surface sensitivity performance of such periodic paired gold nanostructures were evaluated by using the FEM for sensor design based on the LSPRs. From this work, it can be noted that the sensitivity increased as the height and gap between the pair were reduced. For a circular paired disk, the sensitivity was increased when the diameter increased. However, for a paired elliptical disk, the sensitivity increased when the asymmetry increased. In this way, we could achieve a much higher sensitivity with a smaller elliptical disk. Thus, much higher sensitivity can be achieved by using a smaller elliptical antenna pair compared to the much larger circular disks [40]. The strongest transmission dip and absorption peak were achieved at nearly 850 nm for the optimised design, while for other RIs, the plasmonic loss shifted towards a higher wavelength. For a paired elliptical disk with *a*, *b*, *h*, and *g* as 100 nm, 10 nm, 40 nm, and 10 nm, respectively, the sensitivity was calculated as nearly 518–530 nm/RIU, with a 109 nm FWHM and an 8.35654 FOM. The surface sensitivity was also calculated as 240 nm/RIU when the sensing layer was 4.5 nm thick with the refractive index varying from 1.4 to 1.6 to calculate the sensitivity, and the refractive index of the outside domain was 1.33. Additionally, the optimised design can be helpful for the development of future innovative technologies for point-of-care, biomedical, and water quality monitoring systems.

## 5. Novelties and Highlights

- The main novelty of this paper is the demonstration that, by avoiding the circular symmetry, as shown in this manuscript by using elliptical disks, the electric field can be further localised, and this increases the sensitivity of a single elliptical disk;
- Additionally, enhanced field profiles were also shown, which strongly correlates with nearly double the field intensity compared to that of a circular disk, resulting in the sensitivity being enhanced by three-fold;
- The enhancement of the field in the gap of an elliptical dimer and the resultant enhancement of the sensitivity were also demonstrated;
- The fabrication of such an elliptical dimer would be as simple as that of a circular dimer and easier than a ring-shaped dimer. However, the key advantage would be that a similar high sensitivity could be achieved for a much smaller elliptical dimer with less area compared to a circular or ring-shaped dimer of a much larger dimension.

## Figures and Tables

**Table 1:** Comparison of published work with the optimised paired structure.

S. No.	Designed Antenna (nm)	Full-Width at Half-Maximum (nm)	Sensitivity (nm/RIU)	Ref.
1.	Square Shape (*h* = 30, *g* = 30)	125.0985	-	[41]
2.	Disk Shape (*h* = 60, *g* = 14)	147.7624	-	[42]
3.	Bow-Tie Shape (*h* = 90, *g* = 65)	280.4914	-	[43]
4.	Disk Shape (*h* = 40, *g* = 55)	109–113	-	[18]
6.	Nanoshell (*d* = 50)	-	60	[44]
7.	Nanorods, Nanocubes, and Bipyramids	-	195–288	[45,46]
8.	Silver Nanoparticles (*h* = 50)	-	200	[47]
9.	Gold Nanosquare (*h* = 100)	-	167–327	[48]
10.	Nanodisc (*h* = 1)	-	200–350	[49]
11.	Nanotubes (*h* = 100, *g* = 55)	-	250	[50]
12.	Elliptical Antenna	95–100	510–530	Proposed
